# Cardiometabolic Changes in Response to a Calorie-Restricted DASH Diet in Obese Older Adults

**DOI:** 10.3389/fnut.2021.647847

**Published:** 2021-03-19

**Authors:** Cydne A. Perry, Gary P. Van Guilder, Mosharraf Hossain, Alyssa Kauffman

**Affiliations:** ^1^Department of Applied Health Science, Indiana University Bloomington School of Public Health, Bloomington, IN, United States; ^2^High Altitude Exercise Physiology Department, Western Colorado University, Gunnison, CO, United States; ^3^Department of Health and Nutritional Sciences, South Dakota State University, Brookings, SD, United States

**Keywords:** older adults, calorie restriction, DASH diet, obesity, cardiometabolic health

## Abstract

**Objective:** To examine the response of a calorie-restricted Dietary Approaches to Stop Hypertension diet on indicators of cardiometabolic health in a cohort of sedentary obese older adults.

**Design:** This was a controlled-feeding trial with a parallel design. Each participant consumed either 3 oz (85 g; *n* = 15) or 6 oz (170.1 g; *n* = 13) of lean fresh beef within a standardized calorie-restricted DASH-like diet for 12-weeks. Fasted blood samples were collected and used to measure conventional biomarkers of cardiovascular, metabolic and inflammatory health.

**Participants:** Caucasian older (70.8 years), obese (BMI: 32 ± 6.9 kg/m^2^; WC: 101 ± 16.4 cm) females (*n* = 17) and males (*n* = 11) from the rural community of Brookings, South Dakota.

**Results:** 28 participants completed the 12-week feeding trial, with no differences (*p* > 0.05) among the biomarkers of cardiometabolic health between the 3 and 6 oz beef intake groups. However, when the beef intake groups were combined, all biomarkers changed concentration in response to the intervention diet. Total cholesterol (*p* < 0.001), LDL-C (*p* = 0.004), HDL-C (*p* < 0.0001), insulin (*p* = 0.014), glucose (*p* = 0.008), HOMA-IR (*p* < 0.05), IL-12 (*p* < 0.001), and CRP (*p* = 0.006) all decreased in response to the study diet. IGF-1 (*p* < 0.001) and IL-8 (*p* = 0.005) increased in response to the intervention. Correlations among cardiometabolic biomarkers and body composition measures were observed. By study end, the decrease in insulin (*R*^2^ = 0.22; *P* = 0.012) and HOMA-IR (*R*^2^ = 0.22; *P* = 0.01) was positively correlated with the decrease in waist circumference. The increase in IGF-1 was significantly correlated with the decrease in waist circumference (*R*^2^ = 0.21; *p* = 0.014). The increase in IGF-1 was significantly correlated with the increase in sit-to-stand (*R*^2^ = 0.21; *p* = 0.016). The increase in IL-8 was significantly correlated with decreases in total cholesterol (*R*^2^ = 0.24; *P* = 0.008), LDL-C (*R*^2^ = 0.17; *P* = 0.031) and glucose (*R*^2^ = 0.44; *P* = 0.0001).

**Conclusions:** These findings suggest that a DASH-like diet with restricted calories may potentially improve biomarkers of cardiometabolic health in sedentary obese older adults. These results also point to interrelationships between body composition changes and changes in cardiometabolic biomarkers. Lastly, regardless of meat intake amount, positive impacts on cardiometabolic biomarkers were observed in this cohort of older adults with an obese phenotype.

## Introduction

Cardiometabolic disease is an umbrella term that describes a cluster of modifiable risk factors (i.e., hypertension, abdominal adiposity, dyslipidemia, and increased fasting glucose and triglycerides) that increase a person's risk for developing cardiovascular disease, type-2 diabetes, and metabolic syndrome (1, 2). The older adult population is particularly vulnerable to cardiometabolic disease as they are more likely to experience co-existing risk factors (3). Currently, 41% of adults aged 65 years and older in the United States are obese and 80% have at least one chronic disorder related to cardiometabolic disease (4, 5). Furthermore, the older adult population is projected to increase to 98 million by the year 2060 (6). With 47 million Americans experiencing cardiometabolic disorders (7) and the health risks associated with the growing older adult population, it is important to begin implementing targeted intervention strategies that decrease risk factors associated with cardiometabolic disease that will in turn result in the reduction of cardiovascular disease and type-2 diabetes in older adults.

Diet quality is an influential factor in the development of cardiometabolic disease (8, 9) and dietary patterns are vital to the quality of life and survival in older adults (10). Unhealthy diet is one of the leading risk factors for cardiometabolic disease in the United States (11) and accounts for at least 45% of all cardiometabolic deaths (12, 13). With the current diet-related cardiometabolic disease health costs estimated to be $50.4 billion and individual costs being highest among men >65 years (14), effective diet therapies need to be implemented to address this health issue in older adults that in turn reduce the economic burden that ensues. In a 12-week controlled-feeding study examining body composition and muscle strength changes in response to a DASH-like diet in obese older adults, we observed improvements characterized by reductions in body fat, waist circumference, and blood pressure (15). Additionally, handgrip strength was well-maintained with an increase in strength-to-weight ratio (15). Extending the scope of these findings and given the role that abdominal adiposity and blood pressure play on cardiometabolic health, our objectives for this study were two-fold: (i) to evaluate changes in blood biomarkers of cardiovascular, metabolic, and inflammatory health in response to a calorie-restricted DASH study diet in obese adults 65 years and older; and (ii) to assess associations between cardiometabolic biomarkers and body composition measures in this cohort of older adults.

## Materials and Methods

### Study Participants

Participant characteristics, recruitment and study diet were previously reported (15). Briefly, sedentary adults aged 65-years and older were recruited from Brookings, South Dakota from June 2017 to August 2018 (15). Interested volunteers completed a questionnaire that included date of birth, medication use, vitamin and mineral use, and drug and alcohol use prior to the start of the study. Participation on this study depended on the following: (1) age; (2) upward mobile ability; (3) eating one meal per day at the on-site location; (4) not consuming foods and beverages outside of those provided by research personnel; and (5) provide blood samples at 5 timepoints throughout the intervention. A full characterization of body composition measurements and outcomes has been previously published (15). The study was conducted in accordance with the Declaration of Helsinki. The protocol was reviewed and approved by the Institutional Review Board for Human Study Participant Use at South Dakota State University (Approval #: IRB-1712006-EXP) and informed consent was obtained from all participants before entry into the study (ClinicalTrials.gov Identifier: NCT04127240).

### Study Design and Diet Intervention

This was a human controlled-feeding trial with a parallel design in which females (*n* = 17) and males (*n* = 11) aged 65-years and older were assigned to consume either 3 oz (85 g; *n* = 15) or 6 oz (170.1 g; *n* = 13) of lean fresh beef per day within a standardized DASH-like diet as they entered the study. As previously described, the study diet was created using Nutritionist Pro software and based on the DASH eating plan by the National Heart, Lung and Blood Institute, National Institutes of Health (16) and the 2015–2020 *Dietary Guidelines for Americans* for daily caloric intake for older sedentary adults (17). All participants consumed the same standardized DASH-like diet with the exception of the meat intake amounts. The 3 or 6 oz amounts were equally provided among the three major meals: breakfast (1 or 2 oz), lunch (1 or 2 oz), and dinner (1 or 2 oz) for a total of 3 or 6 oz for the entire day. All foods were purchased by research personnel from the local grocery store. All food items were weighed out to the nearest gram and prepared at the South Dakota State University food's laboratory. All participants were required to eat at least one meal per day in the food's laboratory Monday through Friday; all other meals, snacks, and beverages were provided as takeaways. The caloric intake for this study was based upon the 2015–2020 *Dietary Guidelines for Americans* for daily caloric intake for sedentary adults aged 61 years and older (17). The participants that were assigned 3 oz of beef consumed 1,700 calories per day. The 6 oz beef intake group consumed 1,900 calories per day. With beef intake groups combined the participants consumed on average 1,800 calories per day. As previously described, the composition of the study diet included the following estimated (based upon Nutritionist Pro software) daily servings: 7 servings of grains (all whole grains); 5 servings of vegetables; 4 servings of fruits; 3 servings of dairy (low-fat); 4.5 servings of lean meat (average of 3 and 6 oz intakes); 4 servings/week of legumes; 0 servings of sweets. These serving sizes were within the DASH eating plan by the National Heart, Lung and Blood Institute, National Institutes of Health (15, 16). Additionally, as an average of the two meat intake groups, the study diet provided an estimated 1,895 mg/d of sodium, 585 mg magnesium, 4,395 mg potassium, and 1,187 mg calcium, 59% carbohydrates, 21% fat, 20% protein, and 8% saturated fat (15). Since all participants consumed a daily multivitamin/multimineral supplement an additional 50 mg magnesium, 80 mg potassium, and 220 mg calcium was provided.

Since participants were required to eat one meal in the food's laboratory Monday–Friday, investigators had consistent interactions with study participants throughout the study period, which enhanced the compliance to the dietary regimen. In addition, participants verified consumption of each food item by completing a daily checklist provided by the investigators. A multivitamin/multimineral supplement for seniors was provided daily to ensure adequate micronutrient intake.

### Blood Sample Collection and Analytical Measurements

Fasting blood samples were collected in two 10-mL serum separator clot activator tubes (SST Vacutainer; Pulmolab) and two EDTA-coated tubes (Pulmolab) by a trained phlebotomist. The two 10-mL EDTA-coated tubes were put on ice immediately after blood collection and centrifuged within 90 min at 1,055 × g for 15 min at 4°C. The SST tubes were kept at room temperature, allowed to clot, and centrifuged at 650 × g for 15 min at room temperature. All of the samples were aliquoted into 1.8-mL cryostat vials (CryoTube; NUNC) and stored at −80°C.

Quantification of total cholesterol, LDL-cholesterol (LDL-C), HDL-cholesterol (HDL-C), insulin, glucose, insulin-like growth factor 1 (IGF-1), and C-reactive protein (CRP) were performed by the Human Nutritional Chemistry Service Laboratory at Cornell University (Ithaca, NY). The Dimension Xpand plus integrated chemistry automated analyzer (Siemens Healthineers) was used to measure total cholesterol (intra- and interassay CV 1.9 and 8.2%, respectively), LDL-C (intra- and interassay CV 1.3 and 1.0%, respectively), HDL-C (intra- and interassay CV 1.4 and 2.6%, respectively) and glucose (intra- and interassay CV 0.8 and 1.3%, respectively). The Immulite 2000 automated immunoassay system (Siemens Healthineers) was used to measure CRP (intra- and interassay CV 4.5 and 4.1%, respectively), IGF-1 (intra- and interassay CV 4.2 and 7.0%, respectively) and insulin (intra- and interassay CV 5.5 and 3.4%, respectively).

Meso Scale Discovery (Meso Scale Diagnostics, LLC, USA) measured interleukin-8 (intra- and interassay CV 4.7 and 4.4%, respectively) and interleukin-12 (intra- and interassay CV 5.8 and 6.8%, respectively) using the V-plex proinflammatory panel 1 human kit on the Meso QuickPlex SQ 120 with electrochemiluminescence detection.

The homeostatic model assessment of insulin resistance (HOMA-IR) was used to quantify insulin sensitivity using the following formula: fasting plasma glucose (mmol/l) times fasting serum insulin (μIU/mL) divided by 22.5.

### Body Composition and Muscle Strength Measurements

Body composition and muscle measurements were previously detailed and reported (15). Briefly, body mass index was calculated as total body weight in kilograms divided by height in meters squared. A Gulick tape was used to measure abdominal waist circumference. The measurement was taken at the smallest part of the abdomen, above the umbilicus and below the xiphoid process to the nearest 0.1 cm at the end of normal expiration using standard procedures. Total body weight and percent body fat were measured by bioelectrical impedance (InBody 270, InBody USA, Cerritos, California). Handgrip strength (kg) was quantified by the maximum grip force of the right and left hand using a hand-held dynamometer (Smedley III analog). Right and left grip strength data were summed to provide a composite score. Grip strength relative to total body weight was calculated by dividing grip force by the body mass (kg) of the participant at each time point.

### Statistical Analysis

Differences in baseline characteristics between males and females and for beef intake groups were determined by Independent Samples *T*-test. Differences in cardiometabolic and body composition characteristics between beef intake groups at week 12 was determined by Independent Samples *T*-test. Linear mixed models with a random intercept for each participant and Time (weeks 0, 3, 6, 9, and 12) as the fixed effect was used to determine changes in the primary outcome variables across the intervention. An unstructured covariance matrix was assumed. The primary outcome of interest was the difference between baseline and week 12. When indicated by a significant Time effect, pairwise differences at specific time points were identified using the Bonferroni adjustment for multiple comparisons. To adjust for the influence of changes in body weight across the intervention on cardiometabolic variables, we repeated the analyses of the time effect by including body weight as a covariate in linear mixed model. To check the robustness of the primary outcomes, we performed sensitivity analyses with the exclusion of the three normal weight BMI participants. In addition to pooling data for males and females, data are displayed separately by sex. Relations between variables of interest were determined by Pearson's correlation coefficient. Stepwise multiple regression analysis was performed to identify the independent determinants of the change from baseline in wait circumference, IGF-1 levels, and IL-8 levels. In each multiple regression model, variables with a related probability of >0.10 were removed. Statistical significance was set at *p* < 0.05. Data are presented as means (SD) and analyzed with SPSS version 24 (IBM Inc., Armonk, NY, USA).

## Results

### Baseline (Week 0) Characteristics of Study Participants

Twenty-eight participants aged 70.8 years (range = 65–84 years) completed the 12-week controlled-feeding study and were included in the final analysis. Baseline characteristics of cardiometabolic and body composition measures separated by beef intake amounts are presented in Table 1. There were no statistically significant differences (*p* > 0.05) detected between the 3 oz (85 g) and 6 oz (170.1 g) meat intake groups at baseline for age, cardiometabolic biomarkers or body composition measures. Baseline characteristics of cardiometabolic biomarkers separated by sex are presented in Table 2. At baseline, females had statistically higher total cholesterol (*p* = 0.02) and HDL-C (*p* < 0.0001) compared to males. No statistical differences (*p* > 0.05) were detected for LDL-C, insulin, glucose, HOMA-IR, IGF-1, IL-8, IL-12, and CRP between females and males. Baseline body composition characteristics separated by sex has been previously reported (15). Briefly, at baseline males had greater (*p* < 0.05) body fat, waist circumference and grip strength compared to females.

**Table 1:** Baseline characteristics of study participants separated by meat intake group.

**Variables**	**3 oz meat** ** intake group** ** (*n* = 15)**	**6 oz meat** ** intake group** ** (*n* = 13)**	***p*-value**
Age (years)	70.6 (5.9)	71.1 (6.0)	0.8341
Female	8	9	–
Male	7	4	–
**Cardiometabolic markers**			
Total cholesterol (mg/dL)	189.5 (37.3)	171.0 (37.9)	0.2053
LDL-C (mg/dL)	109.4 (29.4)	98.7 (28.2)	0.3364
HDL-C (mg/dL)	53.4 (14.7)	55.0 (19.7)	0.8058
Insulin (μIU/mL)	13.7 (7.0)	13.9 (9.8)	0.9622
Glucose (mg/dL)	105.8 (20.9)	110.2 (26.4)	0.6277
HOMA-IR	3.70 (2.14)	4.24 (4.29)	0.6684
IGF-1 (ng/mL)	96.1 (22.9)	93.3 (17.9)	0.7221
IL8 (pg/mL)	6.20 (2.52)	6.35 (3.18)	0.8887
IL12 (pg/mL)	0.99 (1.12)	0.79 (0.71)	0.7624
CRP (mg/L)	2.69 (2.46)	4.26 (5.36)	0.3173
**Body composition**			
Total body weight (kg)	92.7 (16.8)	87.1 (18.2)	0.4417
Body mass index	32.1 (6.1)	30.4 (6.5)	0.4883
Waist circumference (cm)	100.2 (13.7)	96.0 (14.8)	0.4147
Body fat (%)	36.2 (10.1)	36.2 (10.7)	0.9860
Handgrip strength (kg)	68.4 (22.1)	59.0 (15.2)	0.2032
Sit-to-stand (reps)	11.9 (1.8)	10.5 (2.0)	0.0640

*Data are presented as means and standard deviations with the exception of the number of females and males. Independent samples T-test was performed to determine group differences in the baseline characteristics by meat intake group*.

**Table 2:** Baseline cardiometabolic characteristics separated by sex.

**Variables**	**Females** ** (*n* = 17)**	**Males** ** (*n* = 11)**	***p*-value**
Total cholesterol (mg/dL)	194.3 (36.1)	160.1 (32.3)	**0.02**
LDL-C (mg/dL)	108.3 (31.4)	98.5 (24.9)	0.39
HDL-C (mg/dL)	62.6 (16.2)	41.2 (6.8)	** <0.0001**
Insulin (μIU/mL)	13.3 (9.4)	15.2 (6.4)	0.57
Glucose (mg/dL)	104.7 (23.3)	112.7 (24.0)	0.39
HOMA-IR	3.9 (3.9)	4.3 (2.1)	0.78
IGF-1 (ng/mL)	91.2 (24.0)	100.4 (12.2)	0.25
IL8 (pg/mL)	6.4 (3.4)	6.0 (1.5)	0.73
IL12 (pg/mL)	0.86 (0.6)	1.0 (1.3)	0.71
CRP (mg/L)	3.4 (2.5)	1.8 (1.2)	0.09

*Baseline body composition characteristics separated by sex has been previously reported (15). Data are presented as means and standard deviations. Independent samples T-test was performed to determine group differences in the baseline characteristics between females and males. The bold values indicate the biomarkers that are statistically significant*.

Prior to entry into the study, participants provided information regarding medication use. Self-reported medication use is shown in Supplementary Table 1.

### Cardiometabolic and Body Composition Changes in Response to the Study Diet

Effects of meat intake on cardiometabolic and body composition outcomes at week 12, are presented in Table 3. By week 12 of the intervention there were no statistically significant differences (*p* > 0.05) between the 3 oz (85 g) and 6 oz (170.1 g) meat intake groups on cardiometabolic outcomes or body composition measures.

**Table 3:** Cardiometabolic and body composition characteristics of obese older adults at week 12.

**Variables**	**3 oz meat** ** intake group** ** (*n* = 15)**	**Percent change** ** from baseline**	**6 oz meat** ** intake group** ** (*n* = 13)**	**Percent change** ** from baseline**	***p*-value**
**Cardiometabolic markers**					
Total cholesterol (mg/dL)	176.5 (30.9)	−6.9 (17.2)	164.9 (42.6)	−3.6 (12.4)	0.4203
LDL-C (mg/dL)	104.7 (31.7)	−4.3 (7.8)	94.6 (27.6)	−4.2 (2.1)	0.3796
HDL-C (mg/dL)	49.5 (10.5)	−7.3 (28.6)	49.7 (17.0)	−9.6 (13.7)	0.9757
Insulin (μIU/mL)	10.2 (6.2)	−25.5 (11.4)	11.5 (7.1)	−17.3 (27.6)	0.5994
Glucose (mg/dL)	96.2 (19.2)	−9.1 (8.1)	100.4 (27.5)	−8.9 (4.2)	0.6364
HOMA-IR	2.43 (1.64)	−34.3 (23.4)	3.25 (3.24)	−23.3 (24.5)	0.3961
IGF-1 (ng/mL)	104.1 (21.0)	8.3 (8.3)	100.2 (17.1)	7.4 (4.5)	0.5913
IL-8 (pg/mL)	7.30(4.00)	17.7 (58.7)	7.00 (3.42)	10.2 (7.5)	0.8318
IL-12 (pg/mL)	0.82 (0.98)	−17.2 (12.5)	0.80 (0.65)	1.3 (8.5)	0.9502
CRP (mg/L)	2.76 (2.60)	2.6 (5.7)	3.11 (5.03)	−27.0 (6.2)	0.8159
**Anthropometric measures**					
Total body weight (kg)	86.7 (14.4)	−6.5 (14.3)	82.1 (17.0)	−5.7 (6.6)	0.4495
Body mass index (kg/m^2^)	30.1 (5.8)	−6.2 (4.9)	28.6 (6.0)	−5.9 (7.7)	0.5194
Waist circumference (cm)	96.4 (12.3)	−3.8 (10.2)	92.0 (14.0)	−4.2 (5.4)	0.3815
Body fat (%)	33.4 (11.3)	−7.7 (11.9)	35.0 (9.7)	−3.3 (9.3)	0.7026
Handgrip strength (kg)	68.7 (19.7)	0.4 (10.9)	62.1 (15.1)	5.3 (0.7)	0.3356
Sit-to-stand (reps)	13.9 (2.7)	16.8 (50.0)	13.9 (2.6)	32.4 (30.0)	0.9920

*Data are presented as means and standard deviations. Independent samples T-test on absolute data was performed to determine group differences in the cardiometabolic and body composition characteristics by meat intake group*.

Cardiometabolic changes in response to the intervention diet with both meat intake groups combined are shown in Table 4. Throughout the 12-week intervention period, significant changes in response to the study diet across the intervention period were detected. Significant decreases were observed in all participants within the 12-week intervention period for total cholesterol (*p* < 0.001); LDL-C (*p* = 0.004); HDL-C (*p* < 0.001); insulin (*p* = 0.014); glucose (*p* = 0.008); HOMA-IR (*p* < 0.05); IL-12 (*p* < 0.001) and CRP (*p* = 0.006). Significant increases in response the study diet was detected for IGF-1 (*p* < 0.001); and IL-8 (*p* = 0.005). For all the biomarkers listed in Table 4, observed power for the effect of diet across time was high. Power for the favorable changes across time for total cholesterol, LDL-C, HOMA-IR, IL-12, IGF-1, IL-8, and CRP were >90%. Observed power for glucose was 80 and 75% for insulin. After performing sensitivity analyses with the exclusion of the 3 normal weight BMI participants, it can be concluded that the statistically significant decreases for total cholesterol (*p* < 0.001); LDL-C (*p* = 0.001); HDL-C (*p* < 0.003); insulin (*p* = 0.02); glucose (*p* = 0.0037); HOMA-IR (*p* < 0.0111); IL-12 (*p* < 0.002) and CRP (*p* = 0.02) remained. Sensitively analyses also revealed that the significant increases in IGF-1 (*p* < 0.001) and IL-8 (*p* = 0.007) remained after excluding the normal weight BMI participants. As previously reported, total body weight decreased by 6.3% in all participants by week 12 in response to the intervention diet (15). When adjusting for changes in total body weight, the significant decreases in total cholesterol (adjusted *p* < 0.001), LDL-C (adjusted *p* = 0.002), glucose (adjusted *p* = 0.049) and HOMA-IR (adjusted *p* = 0.045) remained. Changes in body composition measures in response to the intervention diet has been previously reported (15).

**Table 4:** Cardiometabolic biomarker changes in obese older adults consuming the DASH diet for 12-weeks.

	**Weeks of intervention**					
**Variables**	**0**	**3**	**6**	**9**	**12**	***p*-value**
Total cholesterol (mg/dL)	180.9 (38.1)	163.5 (30.7)	164.4 (30.9)	164.2 (34.3)	171.4 (36.3)^*^	<0.001
LDL-C (mg/dL)	104.5 (28.9)	95.7 (25.6)	95.6 (25.6)	96.3 (27.7)^*^	100.0 (29.8)	0.004
HDL-C (mg/dL)	54.2 (16.9)	50.1 (13.8)	49.8 (14.0)	48.0 (13.6)	49.6 (13.6)^*^	<0.001
Insulin (μIU/mL)	14.1 (8.2)	12.4 (8.3)	12.2 (7.7)	10.6 (6.5)	11.3 (6.5)^*^	0.014
Glucose (mg/dL)	108.5 (23.4)	102.4 (14.9)	100.9 (18.6)	98.1 (17.8)	101.3 (20.8)^*^	0.008
HOMA-IR	4.0 (3.3)	3.3 (2.3)	3.3 (2.7)	2.7 (2.2)	3.0 (2.5)^*^	<0.05
IGF-1 (ng/mL)	94.8 (20.4)	103.6 (22.9)	104.7 (21.9)	106.6 (22.1)	102.1 (19.3)^*^	<0.001
IL8 (pg/mL)	6.3 (2.8)	6.7 (4.3)	5.9 (3.2)	9.8 (5.5)^*^	8.1 (4.9)	0.005
IL12 (pg/mL)	0.79 (0.6)	0.65 (0.5)^*^	0.70 (0.5)	0.56 (0.4)^*^	0.69 (0.5)	<0.001
CRP (mg/L)	2.8 (2.2)	1.9 (1.8)^*^	2.3 (2.0)	2.5 (2.5)	2.3 (2.1)^*^	0.006

*Body composition changes in response to the study diet was previously reported (15). Data are presented as means and standard deviations, Linear mixed models with a random intercept for each participant and Time (weeks 0, 3, 6, 9, and 12) as the fixed effect was used to determine changes in the primary outcome variables across the intervention*.

* *p < 0.05 vs. baseline*.

By week 12, in all participants, total cholesterol decreased by 4.9% (*p* < 0.001); HDL-C decreased by 8.5% (*p* < 0.001); insulin decreased by 13.1% (*p* = 0.014); glucose decreased by 8.4% (*p* = 0.008); HOMA-IR decreased by 25% (*p* < 0.05); IGF-1 increased 10% (*p* < 0.001); IL-8 increased by 39% (*p* = 0.005); and CRP decreased by 18% (*p* = 0.006). By week 9, in all participants, LDL-C decreased by 4% (*p* = 0.004) and IL-12 decreased by 12.7% (*p* < 0.001).

At baseline, 46% (8 females; 5 males) of the participants entered the study with features of metabolic abnormalities identified by large waist circumference (males: >40 inches; females >35 inches), low HDL-C (males: <40 mg/dL; females <50 mg/dL), high blood pressure (>130/85 mmHg), and high fasting glucose (>100 mg/dL). As a result of the intervention, by week 12, 17.8% of the participants displayed the above characteristics, representing a significant reduction (*p* = 0.008 for McNemar Chi-square test).

All participants that self-reported medication use at baseline remained on their medications throughout the intervention period except for one male participant whose physician had taken him off of his statin medication.

### Correlations Between Cardiometabolic Biomarkers and Body Composition Measures

**Figure 1:**
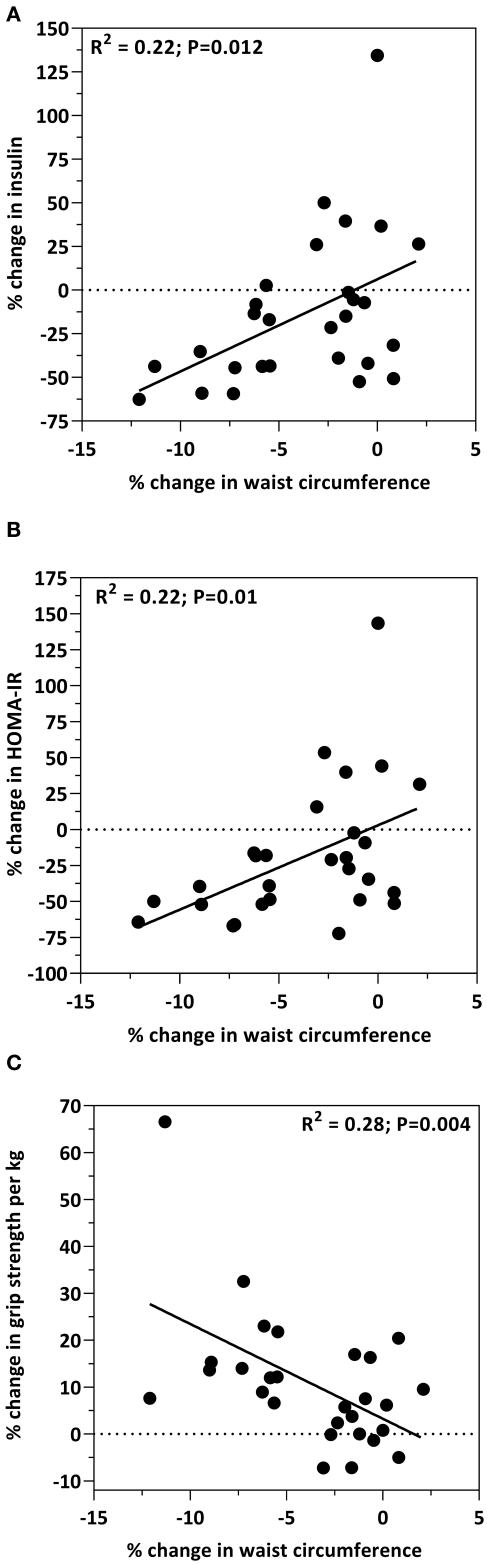
Bivariate correlations between the percent change from baseline in waist circumference and the percent change from baseline in insulin levels **(A)**, HOMA-IR **(B)** and grip strength **(C)**.

Correlations between insulin sensitivity markers and waist circumference are presented in Figure 1. As previously reported, waist circumference decreased by 3.7% in response to the study diet (15). This change in waist circumference was associated with decreases in insulin (*R*^2^ = 0.22; *p* = 0.012; Figure 1A) and HOMA-IR (*R*^2^ = 0.22; *p* = 0.01; Figure 1B). Additionally, there was an inverse relationship between waist circumference and grip strength, such that the decrease in waist circumference was associated with the increase in grip strength (*R*^2^ = 0.28; *p* = 0.004; Figure 1C). Age, and the percent change from baseline in insulin, HOMA, total cholesterol, HDL-cholesterol, LDL-cholesterol, glucose, body weight, percent body fat, BMI, IGF-1, IL-8, IL-12, CRP, grip strength, and 30-s sit-to-stand were included in the multiple regression model to predict the change in waist circumference. The prediction model was statistically significant (*F* = 13.441; *p* < 0.001) and accounted for 50% of the variance of the decrease in waist circumference (Adjusted *R*^2^ = 0.499). Percent reduction in body weight (β = 0.481; *p* = 0.004) and the percent increase in grip strength (β = −0.405; *p* = 0.014) were the only independent predictors of the decrease in waist circumference. All other variables were excluded.

**Figure 2:**
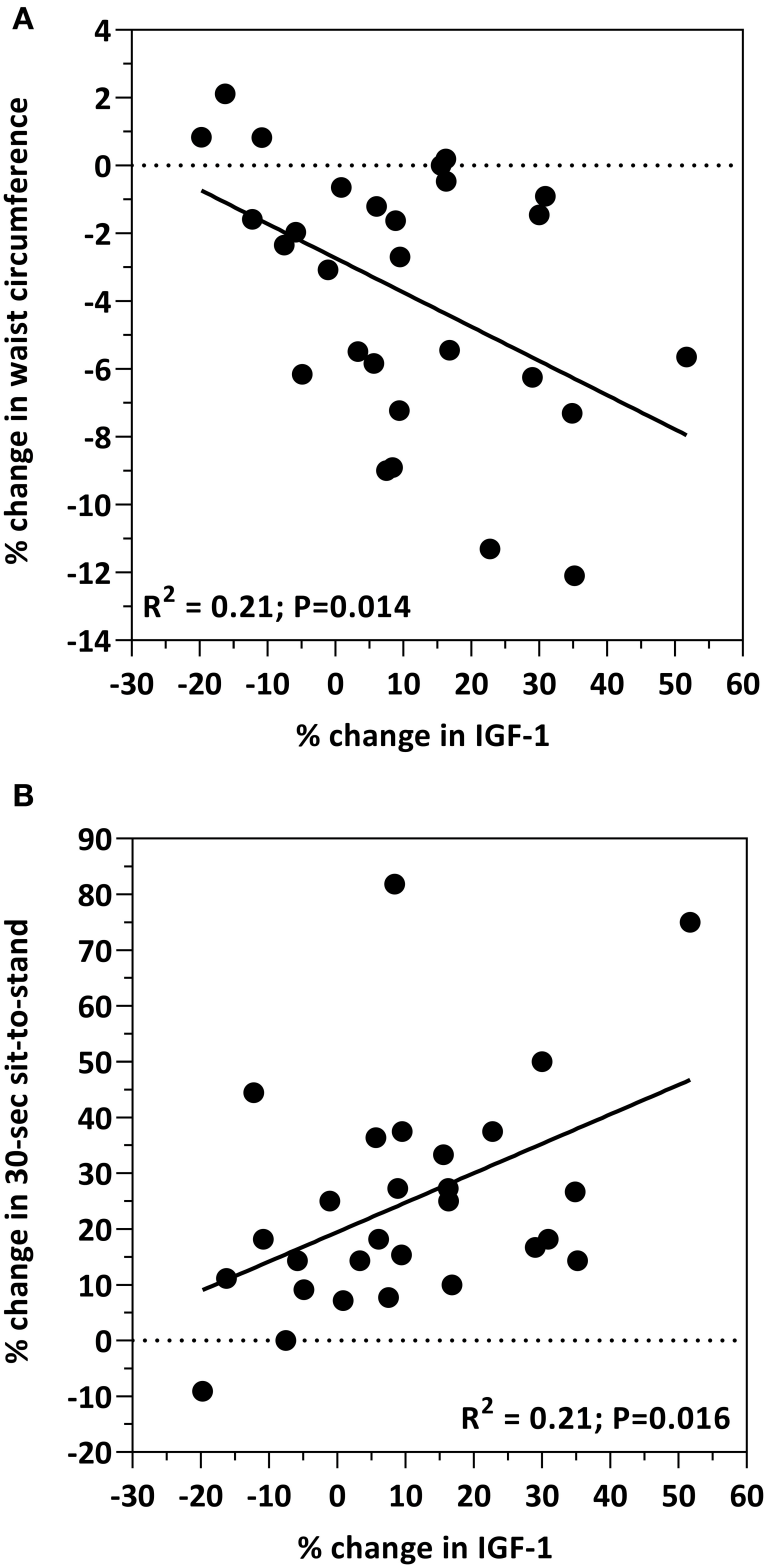
Bivariate correlations between the percent change from baseline in IGF-1 levels and the percent change from baseline in waist circumference **(A)** and the 30-s sit-to-stand test **(B)**.

Associations between IGF-1 and body composition measures are shown in Figure 2. An inverse relationship between IGF-1 and waist circumference was observed, such that the increase in IGF-1 was associated with the decrease in waist circumference (*R*^2^ = 0.21; *p* = 0.014; Figure 2A). The increase in IGF-1 was associated with the increase in the sit-to-stand test (*R*^2^ = 0.21; *p* = 0.016; Figure 2B). Age, and the percent change from baseline in insulin, HOMA, total cholesterol, HDL-cholesterol, LDL-cholesterol, glucose, body weight, percent body fat, BMI, IL-8, IL-12, CRP, grip strength, and 30-s sit-to-stand were included in the multiple regression model to predict the change in IGF-1. The prediction model was statistically significant (*F* = 6.331; *p* = 0.019) and accounted for 18% of the variance of the increase in IGF-1 (Adjusted *R*^2^ = 0.176). Percent increase in the 30-s sit-to-stand test (β = 0.385; *p* = 0.019) independently predicted the increase in IGF-1. All other variables were excluded.

**Figure 3:**
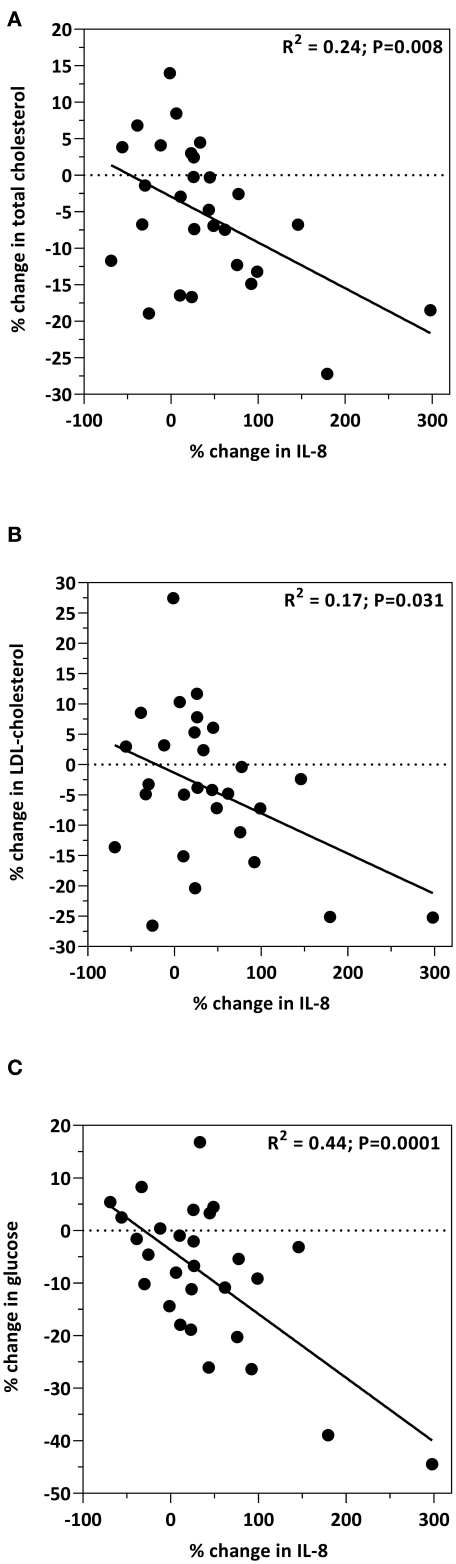
Bivariate correlations between the percent change from baseline in IL-8 levels and the percent change from baseline in plasma total cholesterol **(A)**, LDL-C **(B)** and glucose **(C)**.

Relationships between IL-8 and cardiometabolic biomarkers are presented in Figure 3. The increase in IL-8 was associated with decreases in total cholesterol (*R*^2^ = 0.24; *p* = 0.008; Figure 3A); LDL-C (*R*^2^ = 0.17; *p* = 0.031; Figure 3B); and glucose (*R*^2^ = 0.44; *p* = 0.0001; Figure 3C). Age, and the percent change from baseline in insulin, HOMA, total cholesterol, HDL-cholesterol, LDL-cholesterol, glucose, body weight, percent body fat, BMI, IGF-1, IL-12, CRP, grip strength, and 30-s sit-to-stand were included in the multiple regression model to predict the change in IL-8. The prediction model was statistically significant (*F* = 14.421; *p* < 0.001) and accounted for 52% of the variance of the increase in IL-8 (Adjusted *R*^2^ = 0.518). Percent reduction in glucose levels (β = −0.763; *p* < 0.001) and the percent decrease in IL-12 concentrations (β = 0.327; *p* = 0.033) independently predicted the increase in IL-8. All other variables were excluded.

## Discussion

This highly controlled feeding study sought to evaluate the impact of the DASH diet on changes in biomarkers of cardiometabolic health in a cohort of sedentary obese older adults. Although the results of the present study did not differ between the meat intake groups, there were cardiometabolic changes in response to the 12-week diet intervention on measures of cholesterol, insulin sensitivity, and inflammation when the meat intake groups were combined. Additionally, associations were observed between changes in cardiometabolic biomarkers and changes in body composition measures.

### In Response to the DASH Diet, Total Cholesterol, LDL-C, and HDL-C Decreased in Obese Older Adults

In the present study under controlled-feeding intakes, total cholesterol was significantly reduced in all participants by 4.9% (*p* < 0.001) and LDL cholesterol was reduced by 4% (*p* = 0.004). There were no differences in cholesterol outcomes when separated by meat intake groups. The study diet provided on average an estimated 195.5 mg of dietary cholesterol per day that was consistently consumed throughout the 12-week intervention period. A review by Grundy, showed that several metabolic studies have reported a linear relationship between dietary cholesterol intake and serum cholesterol levels (18). In fact, Keys et al. (19) showed that a dietary cholesterol intake of about 200 mg per day should result in a decrease in blood cholesterol levels by 5%, an observation shown by the present study in a cohort of obese older adults. The DASH dietary pattern does not include recommendations for dietary cholesterol intake and the 2015–2020 *Dietary Guidelines for Americans* no longer recommends limiting dietary cholesterol intake to 300 mg per day. Dietary cholesterol, however, is not the only factor that influences serum cholesterol concentrations. Saturated fat, when consumed above current recommendations, also affects blood cholesterol and saturated fat has a greater negative impact on the development of cardiovascular disease (20). As previously reported 8% of the total calories from the study diet was saturated fat (15), which is within the 2015–2020 *Dietary Guidelines for Americans* to limit saturated fat intake to <10%, but above the American Heart Association recommendations of <7%. Cholesterol and saturated fat, however, are only two components of a whole-diet and other factors, such as total calories and dietary fiber need to be considered with changes in cholesterol levels. The diet for the present study provided on average 1,800 calories per day as recommended by the USDA for sedentary older adults (15, 17). At 1,800 calories, the DASH diet recommends substantial amounts of whole-grains, fruits and vegetables, all of which the study diet provided (15, 16). Adhering to these recommendations, the study diet provided 29.9 g of total dietary fiber, which is a major dietary factor that aids in further lowering cholesterol levels. The cholesterol lowering benefits of the DASH diet have been previously reported (21) and findings from the present show similar results. Due to the well-established cardiovascular benefits of the DASH dietary pattern and cardiovascular disease remaining as the number one cause of death in the United States, more aggressive implementation strategies may need to be established to begin a population-wide adherence to the DASH diet.

Previous reports have documented that the DASH diet lowers HDL cholesterol (HDL-C) (21–24). In the present study, HDL-C decreased by 8.5% (*p* < 0.001) from baseline to study-end in all participants; no differences in HDL-C levels were observed based upon meat intake groups. In a cohort of middle-aged overweight adults, Chiu et al. compared the DASH diet (27% total calories from fat) to a high-fat DASH diet (HF-DASH; 40% total calories from fat) in which HDL-C decreased with consumption of the DASH diet (24). This outcome was not observed with consumption of the HF-DASH diet. Similarly, the diet for the present study provided 21% total calories from fat (15) and the outcomes were similar in this cohort of obese older adults. Although it has been previously reported that HDL-C concentrations drop with consumption of a low-fat diet (25), it is unknown of the impact of the DASH diet on functional changes to HDL. There are several functions of HDL-C that are vital for cardioprotection such as reverse cholesterol transport, anti-inflammatory action, modulation of glucose metabolism, and endothelial protection (26). Although HDL-C concentrations decreased in the present study, it is unknown whether this decease impacted HDL-C functionality that in turn may have negatively impacted cardiovascular health. To gain a better understanding of this relationship, future DASH diet intervention studies should include measures of HDL-C functionality and ascertain the impact on cardiovascular health.

### Markers of Insulin Sensitivity Improved and Were Associated With Reductions in Abdominal Adiposity in Older Adults With an Obese Phenotype Consuming the DASH Diet

Outcomes of the present study show that consumption of the study diet for 12-weeks, under controlled feeding conditions, among a cohort of obese older adults resulted in reductions in insulin, glucose and HOMA-IR. Insulin was significantly reduced in all participants by 13.4% (*p* = 0.014) and glucose was reduced by 8.4% (*p* = 0.008). Furthermore, HOMA-IR decreased by 25% (*p* < 0.05) by study end. Although recent systematic reviews have reported that the DASH diet has no beneficial effect on fasting blood glucose and HOMA-IR (27, 28), similar findings of the present study have been reported among type 2 diabetics and women with gestational diabetes (29–31). Shirini et al. concluded that the DASH diet may improve insulin sensitivity independent of weight loss (27). We previously reported that the participants in the present study reduced total body weight by 6.3% (15). After adjusting for changes in total body weight, the significant decreases in glucose (adjusted *p* = 0.049) and HOMA-IR (adjusted *p* = 0.045) remained.

One modifiable risk factor associated with cardiometabolic disease is abdominal adiposity (2). Abdominal adiposity is the hallmark of the obese phenotype in older adults and is the primary contributing factor to chronic disease risk. Waist circumference serves as a measure of abdominal adiposity and a surrogate indicator of cardiometabolic disease risk (32, 33). We previously reported a 3.7% reduction in waist circumference in this cohort of older adults and that this reduction may in part be due to the decrease in body fat as a result of the intervention (15). In the present study we report that the decrease in waist circumference is correlated with the decrease in insulin (*R*^2^ = 0.22; *p* = 0.012; Figure 1A) and HOMA-IR (*R*^2^ = 0.22; *p* = 0.01; Figure 1B). The association between abdominal adiposity and insulin resistance is well-established, specifically in populations with obesity and type 2 diabetes (34–36) as well as healthy populations (e.g., women and adolescents) (37, 38). Interestingly, Díez-Fernández et al. recently reported that in young adults, waist circumference mediates the relationship between muscular strength and cardiometabolic risk (39). What is important to appreciate is that increased abdominal adiposity results in a redistribution of ectopic adipose tissue within skeletal muscle. Ectopic fat deposited in skeletal muscle contributes to poor skeletal muscle function characterized by reduced muscle mass and strength, and impaired glucose tolerance. This is crucial given that skeletal muscle is the largest consumer of glucose and plays a central role with insulin sensitivity. Indeed, the outcomes of the present study show that the decrease in waist circumference was inversely related to muscle strength (*R*^2^ = 0.28; *p* = 0.004; Figure 1C) suggesting a favorable change in skeletal muscle function may be the result of reduced abdominal obesity. Although previous reports as well as the outcomes from the present study show that an interrelationship between insulin sensitivity, abdominal adiposity, and muscle health exists, more studies are needed in various populations to better understand the role that diet, dietary patterns or dietary components play within this interrelationship.

Insulin like growth factor-1 (IGF-1), a growth hormone produced by the liver, exerts its effects on glucose regulation and is positively correlated with insulin sensitivity and muscle health (40, 41). For example, IGF-1 has several anabolic properties (i.e., cell growth and differentiation, mitochondrial biogenesis, reduced inflammation, neuromuscular junction stability) on skeletal muscle that counteract the development of sarcopenia by activating AMPK and PGC1α. Low IGF-1 concentrations are associated with several cardiometabolic risk factors including obesity, insulin resistance, diabetes and inflammation (40–45). Low circulating IGF-1 levels may also predict for increased risk of heart disease and myocardial infarction (46–48). Conversely, increased IGF-1 levels are paralleled with improvements in insulin sensitivity in premenopausal obese women with insulin resistance consuming a calorie-restricted diet (49). In the present study, IGF-1 concentrations increased by 10% (*p* < 0.001) in all participants as a result of the intervention. Moreover, this increase was negatively associated with waist circumference (*R*^2^ = 0.21; *p* = 0.014; Figure 2A), a similar finding reported by Succurro et al. in a cohort of non-diabetetic adults (50). Although several clinical studies report associations between IGF-1 and muscle strength in various human populations (e.g., healthy adults, older women and sarcopenic obese elderly) (51–53), they primarily focused on aging and/or exercise. In the present study, in which the focus was diet, we observed a positive relationship between IGF-1 and muscle strength (*R*^2^ = 0.21; *p* = 0.016; Figure 2B). While these findings collectively are suggestive of a relationship between diet, IGF-1, abdominal adiposity and muscle, this area remains highly unexplored and more studies are required to elucidate these relationships and impact on cardiometabolic outcomes.

### Inflammatory Biomarkers Were Influenced by Consumption of the DASH Diet in Older Adults With an Obese Phenotype

Interleukin 8 (IL-8) is a chemokine involved in ischemic tissue repair and thought to exert beneficial effects on cardioprotection (54). Circulating IL-8 concentrations, however, are elevated in obese individuals and are considered to be a factor relating obesity to increased cardiovascular risk (55). Results of the present study, in a cohort of obese older adults showed that by week 9 of the intervention, IL-8 levels increased by 38.8% (*p* = 0.005) as a result of the study diet. Most notably, this increase was associated with decreases in total cholesterol (*R*^2^ = 0.24; *p* = 0.008; Figure 3A), LDL-C (*R*^2^ = 0.17; *p* = 0.031; Figure 3B), and glucose (*R*^2^ = 0.44; *p* = 0.0001; Figure 3C), pointing toward a more cardioprotective role for IL-8. Reports of circulating concentrations of IL-8 on heart function have resulted in conflicting outcomes. A case study investigating serum levels of IL-8 on myocardial infarction (MI) showed that concentrations were associated with increased MI risk in men, but reduced occurrence of MI in women (56). In rodents treated with IGF-1, increased IL-8 displayed a proangiogenic effect with protection against ischemic myocardium (57). Indeed, the concentrations of IGF-1 in the present study increased by 10% (*p* < 0.001). Although previous reports showed an association of IL-8 with BMI, fat mass, and waist-to-hip ratio in obese individuals (55), the results of the present study did not observe such associations. With conflicting outcomes from previous reports, including the present study, many more studies are required to fully uncover the role of IL-8 in heart health, specifically with regard to cardioprotection and risk of cardiovascular disease. Future studies are needed to determine specific populations that benefit from increased IL-8 and which populations are negatively affected.

Interleukin 12 (IL-12) is a proinflammatory cytokine involved in the pathogenesis of numerous inflammatory disorders such as psoriasis, crohn's disease, ulcerative colitis, multiple sclerosis, and rheumatoid arthritis (58). IL-12 production is a contributing factor in obesity-related inflammation and insulin resistance (59). In rodent studies involving older mice, treatment with an anti-IL-12 monoclonal antibody alleviated the inflammatory bowel disease, colitis (60). Additionally, mice consuming a low-carbohydrate, soy, fish oil diet showed reduced IL-12 concentrations in bronchial tissue, leading to decreased inflammation and DNA damage in the lungs (61). Indeed, results of the present study showed that in response to the study diet circulating IL-12 levels decreased by 12.7% (*p* < 0.001) by week 9. Although these findings are suggestive of an improved inflammatory state or reduced risk for the onset of IL-12 induced disorders, more human studies are required to determine whether diet-induced reductions in IL-12 prevent or alleviate inflammatory disorders in which IL-12 has a role.

In the present study C-reactive protein (CRP) decreased by 11.3% (*p* = 0.006) in all participants over the course of the intervention. CRP is a widely used biomarker of inflammation and is elevated in individuals with an obese phenotype (62). When elevated, it serves as a marker for insulin resistance (63) and is associated with coronary artery disease and total mortality (64, 65). CRP may also serve as a possible biomarker for infection and pneumonia in geriatric patients (66). Furthermore, CRP has been associated with HOMA-IR and inversely related with grip strength (63, 67). Indeed, HOMA-IR decreased (*p* < 0.05) in the present study and strength-to-weight ratio increased as previously reported (15). A recent meta-analysis reported that the DASH diet had no effect on CRP concentrations (28). However, consumption of the DASH diet among type-2 diabetics reduced CRP levels by 26.9% (68) and the DASH diet was associated with a reduction in CRP in women (69). It is possible that the DASH diet may not be effective in reducing CRP levels in healthy adults, but rather in individuals with obesity and/or diabetes.

## Limitations

Limitations of this study include the following: (i) a non-intervention control group was not included in this study; (ii) cohort of participants in the present study were all white which is representative of the dominant racial background in the state of South Dakota; (iii) all participants were upwardly mobile and lived in their own homes; (iv) no participants required support for daily living activities; no one resided in assisted living facilities; (v) overall, regardless of removing the three participants with a normal weight BMI significant differences in the cardiometabolic responses remained. Due to these limitations great caution should be taken when generalizing the outcomes of the present study to various populations of older adults with diverse ethnic/racial and demographic backgrounds as well as different living conditions.

## Conclusions

Results from the present study confirm that the DASH diet with restricted calories beased upon the U.S. Dietary Guidelines for Americans is an effective approach to improve blood biomarkers of cardiovascular, metabolic, and inflammatory health in obese older adults. The outcomes of the present study also show positive cardiometabolic improvements with daily lean beef consumption. Because older adults are especially vulnerable to cardiometabolic disease and unhealthy diet is a leading risk factor for the development of cardiometabolic disease strategies for dietary behavioral change may be need implemented to increase the adoption of the DASH diet.

## Data Availability Statement

The original contributions presented in the study are included in the article/Supplementary Material, further inquiries can be directed to the corresponding author/s.

## Ethics Statement

The studies involving human participants were reviewed and approved by the Institutional Review Board for Human Study Participant Use at South Dakota State University (Approval #: IRB-1712006-EXP). The study was conducted in accordance with the Declaration of Helsinki and informed consent was obtained from all participants before entry into the study. The patients/participants provided their written informed consent to participate in this study.

## Author Contributions

CP: conceptualization/study design, methodology, manuscript preparation, review, editing, supervision, project administration, and funding acquisition. GV: statistical analysis, review, and editing. MH and AK: data curation and review. All authors contributed to the article and approved the submitted version.

## Conflict of Interest

The authors declare that the research was conducted in the absence of any commercial or financial relationships that could be construed as a potential conflict of interest.
