# Supplementary material for: Cardiometabolic Changes in Response to a Calorie-Restricted DASH Diet in Obese Older Adults
Source: Front Nutr. 2021 Mar 19;8:647847. doi: 10.3389/fnut.2021.647847 (PMC8017169; doi:10.3389/fnut.2021.647847)
Supplement: Supplementary file 1 [file Table_1.DOCX]

**Supplemental Table 1:** Self-reported medication use by study participants

| Medication Name | Number of Participants Taking Medication |
| --- | --- |
| Lisinopril | 3 |
| Hydrochlorothiazide | 1 |
| Statins | 6 |
| Ibandronate sodium | 1 |
| Montelukast sodium | 1 |
| Fluoxetine HCl | 1 |
| Metoprolol | 3 |
| Omeprazole | 1 |
| Loratidine | 1 |
| Nasacort | 1 |
| Plavix | 1 |
| Tamsulosin | 1 |
| low dose aspirin | 4 |
| Venlafaxine | 1 |
| Losartan Potassium | 1 |
| Meloxicam | 1 |
| Lantus | 1 |
| Victoza | 1 |
| Glyburide | 1 |
| Sertraline | 1 |
| Metformin | 1 |
| Glipzide | 1 |
| Farxiga | 1 |
| Xarelto | 1 |
| Pantoprazole | 1 |
